# SARS-CoV-2 variant N.9 identified in Rio de Janeiro, Brazil

**DOI:** 10.1590/0074-02760210166

**Published:** 2021-11-08

**Authors:** Luis Fernando Lopez Tort, Ieda Pereira Ribeiro, Lidiane Souza Raphael Menezes, Alexandre Araújo Cunha dos Santos, Marta Pereira Santos, Luana Damasceno, Paola Cristina Resende Silva, Marilda Agudo Mendonça Teixeira de Siqueira, Patricia Brasil, Myrna Cristina Bonaldo

**Affiliations:** 1 Fundação Oswaldo Cruz-Fiocruz, Instituto Oswaldo Cruz, Laboratório de Biologia Molecular de Flavivírus, Rio de Janeiro, RJ, Brasil; 2 Universidad de la República, Centro Universitario Regional Litoral Norte, Laboratorio de Virología Molecular, Salto, Uruguay; 3 Fundação Oswaldo Cruz-Fiocruz, Instituto Nacional de Infectologia Evandro Chagas, Rio de Janeiro, RJ, Brasil; 4 Fundação Oswaldo Cruz-Fiocruz, Instituto Oswaldo Cruz, Laboratório de Vírus Respiratórios e do Sarampo, Rio de Janeiro, RJ, Brasil

**Keywords:** SARS-CoV-2, COVID-19, variants of interest, lineage N.9, E484K, Rio de Janeiro, Brazil

## Abstract

**BACKGROUND:**

The severe acute respiratory syndrome coronavirus 2 (SARS-CoV-2) B.1.1.33-derived lineage named N.9 was described recently in Brazil and it’s considered a potential variant of interest (VOI) due to the presence of E484K substitution at the receptor-binding domain (RBD) of the Spike (S) protein.

**OBJECTIVE:**

To describe the first detection of variant N.9 in Rio de Janeiro State.

**METHODS:**

SARS-CoV-2 N.9 was confirmed by quantitative reverse transcription polymerase chain reaction (qRT-PCR), whole-genome sequencing and phylogenetic analysis.

**FINDINGS:**

Here, we report two SARS-CoV-2 N.9 lineage strains in Rio de Janeiro. One of them had only the E484K substitution of the six N.9 lineage-defining mutations. Other three strains pre-defined as N.9 have the same genomic profile. These four strains are grouped within the B.1.1.33 lineage and basal to the N.9 lineage in our phylogenetic analysis, and we call them “N.9-like/B.1.1.33 + E484K”.

**MAIN CONCLUSIONS:**

The phylogenetic analysis shows four independent introductions of N.9 in the state of Rio de Janeiro in October and December 2020, January and March 2021. SARS-CoV-2 N.9 dissemination in the Rio de Janeiro could have been limited by the emergence and dominance of other variants, mainly by the lineage P.2 VOI Zeta that emerged in the same period and co-circulated with N.9, as observed in the neighboring State of São Paulo.

The severe acute respiratory syndrome coronavirus 2 (SARS-CoV-2) that causes the coronavirus disease 2019 (COVID-19) pandemic has reached every continent since December 2019.^1^ A novel variant of the virus emerged at the end of January 2020 in lineage B.1 presented the spike protein substitution D614G. This variant replaced the initial strain identified in Wuhan (Wuhan-Hu1) and became the globally dominant strain of SARS-CoV-2.^2^ ^,^ ^3^ Since then, several circulating variants of SARS-CoV-2 have raised significant concerns about the impact on viral fitness, transmissibility, pathogenicity, and immunological escape. The first wave of the COVID-19 epidemic in Brazil in 2020 was dominated by the presence of B.1.1.28 and B.1.1.33 lineages.^4^ ^,^ ^5^ In Rio de Janeiro, the B.1.1.33 reached the dominance of 80% in the early pandemic phase.^5^ A second and most significant wave of COVID-19 epidemic in Brazil started in December 2020 in the Amazonas State, and it was associated with the emergence of the variant of concern (VOC) P.1 and described as the prevalent SARS-CoV-2 lineage detected in this state.^6^ ^,^ ^7^ By March 2021, the lineage P.1 was detected as the most prevalent lineage all across the country (http://www.genomahcov.fiocruz.br, accessed on May 2nd, 2021). On the other hand, the lineage P.2 VOI Zeta, probably emerged in Brazil in July 2020,^8^ ^,^ ^9^ and has been detected as the most prevalent variant in several states across the country until late 2020 and early 2021 (http://www.genomahcov.fiocruz.br, accessed on May 2nd, 2021). Both lineages have emerged from lineage B.1.1.28. In the same way, at least two B.1.1.33-derived lineages have recently emerged in Brazil: N.9^10^ and N.10.^11^ The lineage N.9 probably emerged in August 2020 in the State of São Paulo, has spread across different Brazilian states (Southeast, South, North, and Northeast regions), and comprise a high fraction (35%) of the B.1.1.33 sequences detected between November 2020 and February 2021. The lineage N.9 is also considered a potential VOI due to the presence of E484K amino acid substitution at the receptor-binding domain (RBD) of the Spike (S) protein.^10^ This mutation has been described as one of the most important immune evasion mechanisms, as it confers resistance to several monoclonal antibodies and reduces the neutralisation effect of sera obtained from convalescent and vaccinated individuals.^12^ ^,^ ^13^ ^,^ ^14^

## MATERIALS AND METHODS

**Fig. 1:**
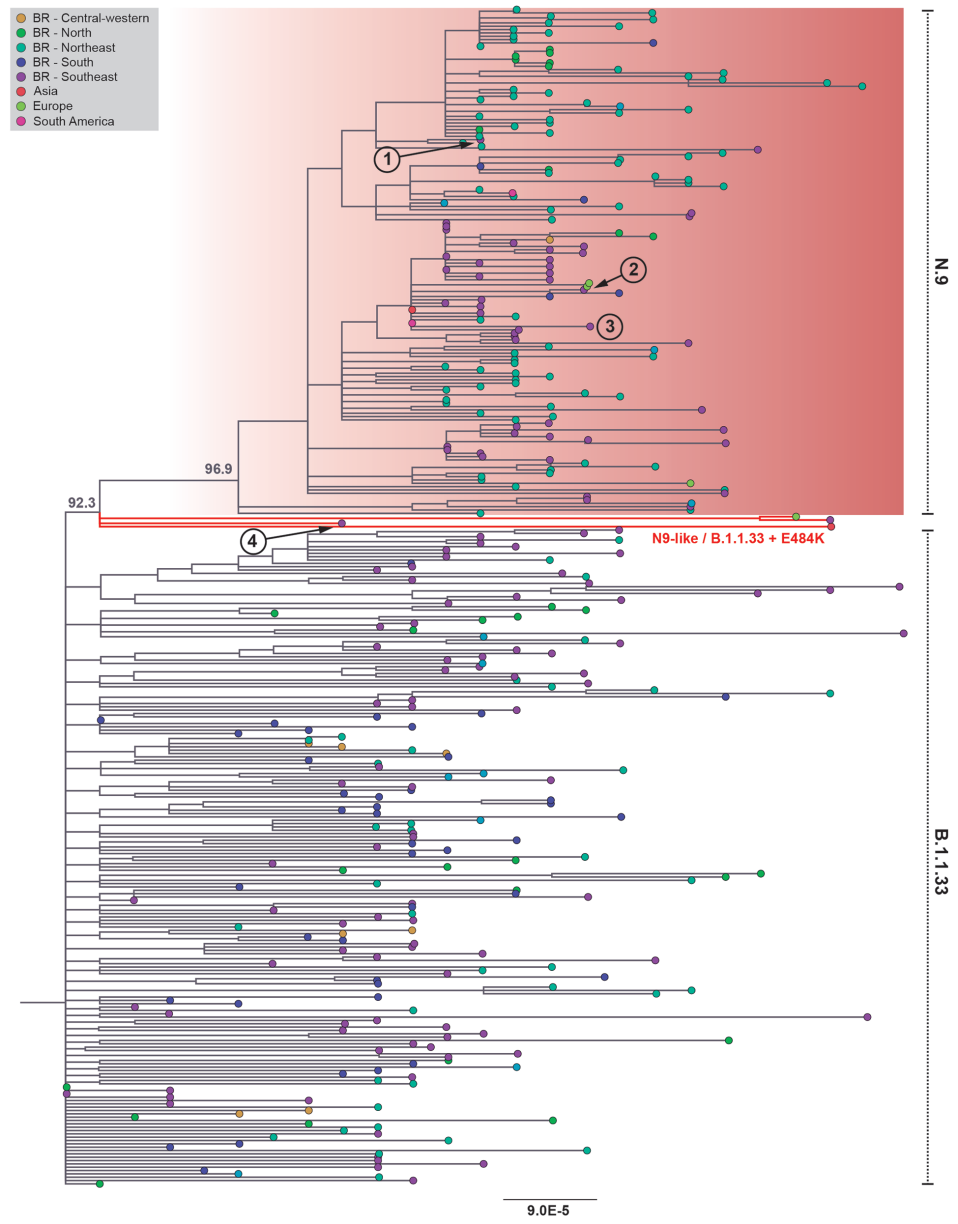
maximum likelihood phylogenetic analysis of severe acute respiratory syndrome coronavirus (SARS-CoV) N9 and N9-like sequences. Maximum likelihood phylogenetic tree (*n* = 353) of whole-genome sequences (*n* = 29,611 nts) of SARS-CoV-2 inferred with the N.9 genomes identified in this study (n = 2) in addition to B.1.1.33 (*n* = 197) and all available sequences of the variant of interest (VOI) N.9, including the two query sequences, (*n* = 156) by August 13th, 2021 at the EpiCoV database of the GISAID initiative. The origin of all sequences is indicated by their tip colors according to the legend in the upper left corner. The N9 phylogenetic cluster is highlighted in red, and both, B.1.1.33 and N.9, have their limits indicated by a dotted line on their right. Branches leading to the four “N.9-like / B.1.1.33 + E484K” genomes are also coloured in red. The statistical support (SH-aLRT) of relevant nodes in the tree is annotated. Divergence in the tree is represented in accordance with the scale in the bottom. The position of the four N.9 strains identified in the state of Rio de Janeiro are numbered (1 - 4), their GISAID ID being: (1) EPI_ISL_2086268, (2) EPI_ISL_2249431, (3) EPI_ISL_2629682, and (4) EPI_ISL_2557401.

In the present study, we described the first detection of N.9 in the State of Rio de Janeiro. The genome of this N.9 strain was detected in two clinical specimens, nasopharyngeal swab and saliva, collected on December 9th, 2020, from a 32 years old diabetic patient, male, resident in the city of Rio de Janeiro. The patient had no history of traveling in the last two months. He reported self-limited fatigue, myalgia, cough, headache, and mild fever during five days, with no loss of taste or smell and no need for hospitalisation. SARS-CoV-2 infection was confirmed by real time RT-PCR by using the 2019-nCoV RUO Kit (Integrated DNA Technologies, Coralville, USA), two days after the symptoms onset (December 9th) in the nasopharyngeal swab and saliva clinical specimens. The subject provided written, informed consent before participation in the study, and a medical assistant filled a standardised medical questionnaire form during an interview with the participant. The institutional review boards at Fundação Oswaldo Cruz (FIOCRUZ) and National Research Ethics Commission approved the study protocol (CAAE: 30639420.0.0000.5262/032458/2020). The SARS-CoV-2 whole-genome nucleotide sequencing was generated with a RT-PCR scheme containing 25 overlapping amplicons of 1000 to 1500 bp in length covering the entire genome (Supplementary data I - Tables I-II). The DNA sequencing was performed at the sequencing facility of Fiocruz-RJ (RPT01A - Sequenciamento de DNA - RJ). Sequences were edited and assembled to obtain the whole-genome consensus sequence using the SeqMan Pro version 8.1.5 software (DNASTAR, Madison, USA) and the complete genome sequence of Wuhan Hu-1 (MN908947.3) as reference. The whole-genome SARS-CoV-2 consensus sequence was initially classified as N.9 according to the PANGO Lineage web application (https://pangolin.cog-uk.io),^3^ and it was later confirmed through a phylogenetic analysis (Fig. 1). Three other N.9 recognised by the Pango Lineage were later detected in Rio de Janeiro, one of them by our research group. This N.9 genome detected in the present study was generated at the Laboratory of Respiratory Viruses and Measles, FIOCRUZ, using an in-house methodology as previously described.^9^ All available N.9 (n = 156) and all Brazilian B.1.1.33 genomes (n = 1458) by August 13th, 2021 in EpiCoV database in the GISAID (https://www.gisaid.org/) were downloaded (Supplementary data II and III). To preserve B.1.1.33 diversity but also limit its dimension, a downsampling was done with CD-HIT v.4.8.1.^15^ The final dataset (*n* = 353) was aligned by using MAFFT online service v.7.^16^ The alignment was manually edited by using AliView software v.1.26^17^ and subjected to a maximum likelihood (ML) phylogenetic analysis with IQ-TREE v2.1.3,^18^ using a GTR + I + F + Г4 nucleotide substitution model as selected by ModelFinder.^19^ The reliability of the obtained tree topology was estimated with the approximate likelihood-ratio test.^20^ The ML tree generated was visualised by using FigTree v.1.4.4 (http://tree.bio.ed.ac.uk/software/figtree/). The mutational profile was investigated using the Nextclade v.0.14.2 (https://clades.nextstrain.org, Nextclade 2020-2021 Nextstrain developers), and the CoVSurver mutations App (https://www.gisaid.org/epiflu-applications/covsurver-mutations-app/, A*STAR Bioinformatics Institute, Singapore).

## RESULTS AND DISCUSSION

**Fig. 2:**
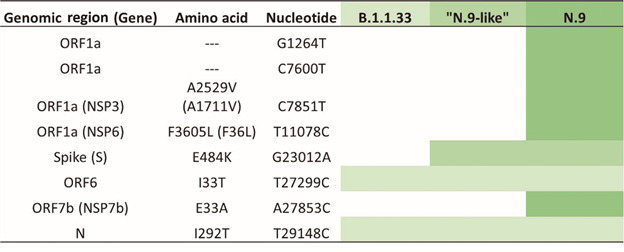
evolutionary steps associated with the possible emergence of N.9 and “N.9-like / B.1.1.33 + E484K” lineages. Each colored line represents a mutation that emerged during the diversification of B.1.1.33 lineage in Brazil originating the N.9 and “N.9-like / B.1.1.33 + E484K”.

The six synapomorphic mutations of SARS-CoV-2 lineage N.9^10^ were confirmed in the first one of the N.9 genomes obtained in this study (EPI_ISL_2086268) and it grouped in the N.9 cluster in our phylogenetic analysis (Fig. 1, upper part). The genome of SARS-CoV-2 from saliva could not be recovered, although several attempts were made to amplify it through RT-PCR SARS-CoV-2 whole-genome protocol. Perhaps, it was due to the low viral load of the sample as determined by the high Ct value obtained at the real time RT-PCR (Ct = 31,02). The other one (EPI_ISL_2557401), assigned by PANGO Lineage as N.9 lineage, only has one of the six synapomorphic mutations: the G23012A, S: E484K (Fig. 2). Other three strains defined as N.9 in the EpiCoV database at GISAID have the same genomic profile. These four strains are grouped within the B.1.1.33 lineage and basal to the N.9 lineage in our phylogenetic analysis (Fig. 1, middle part). They seem to be another example of convergent evolution of the E484K mutation in different lineages, and we call them “N.9-like / B.1.1.33 + E484K”. This convergent evolution of E484K can be evidenced by its appearance multiple times independently around the world in four variants: VOC Beta, VOC Gamma, VOC Kappa and VOI Zeta.^8^ ^,^ ^21^ By phylogenetic reconstruction the four N.9 genomes described in Rio de Janeiro were four independent introductions in the State of Rio de Janeiro in October and December 2020 and January and March 2021.

There is no evidence of the clinical impact of N.9 in the literature.^10^ And, in the same way, by analysing the metadata associated with each of the 138 genomes available in the EpiCoV database at GISAID (by June 28th), only 24% (n = 33) of them have epidemiological information without clinical symptoms description. From these data we observed the next epidemiological information: (i) the patients age range were from 16 to 90 years old, with a media age of 50.63 years old, (ii) 60% of the cases were from patients older than 60 years old, (iii) four patient died (all males), and three of them were older than 75 years old, the other were a patient of 51 years old, and (iv) 18/33 were male, and 15/33 were female. There is no information in the EpiCoV database at GISAID related to treatment, hospitalisation requirement or clinical symptoms.

**Fig. 3:**
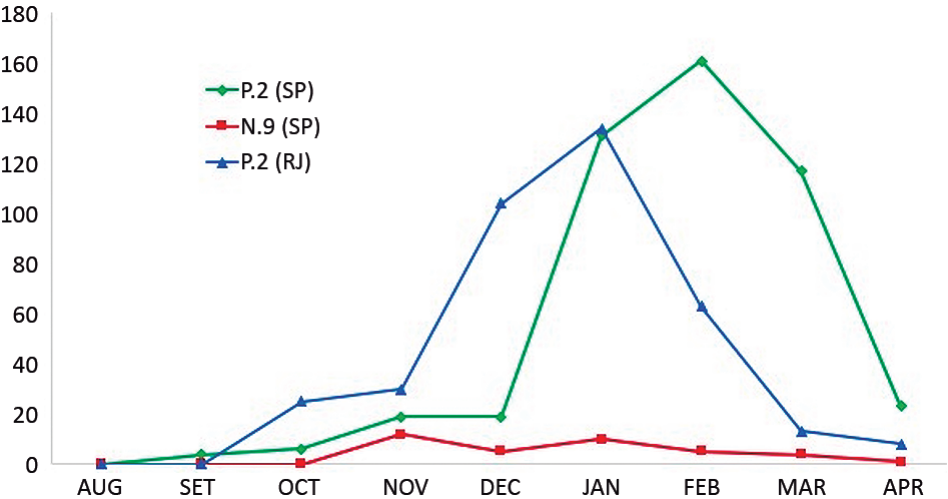
number of P.2 and N.9 genomes detected in São Paulo and Rio de Janeiro states between August/2020 and April/2021.

The plot of the frequency of P.2 and N.9 lineages that co-circulated in São Paulo State, and P.2 in Rio de Janeiro, between August/2020 and April/2021 (Fig. 3), allows us to hypothesise that probably there was a competition between N.9 and P.2 lineages in these states, and it seems that P.2 was able to quickly spread in these populations, and, this way, limited the N.9 dissemination in the same populations. Both lineages have the same unique beneficial mutation (E484K) and are very similar genotypically. So, we think that other factors external to viral fitness allowed P.2 to be prevalent over N.9, for example the population behavior regarding non pharmaceutical interventions at the moment of emergence and introduction of this lineage. In this sense, an important fact to be considered is that P.2 emerged earlier than N.9. The first genome of P.2 strain identified in Brazil, as accessed in the EpiCoV database at GISAID, was collected in April/2020, and, on the other hand, the first genome of N.9 was collected in October/2020.

In conclusion, we report the first genome description of the N.9 lineage in the state of Rio de Janeiro, where the SARS-CoV-2 VOC Alpha, VOC Gamma and the VOI Zeta also circulated. We also described an apparently B.1.1.33-derived sublineage that we called as “N.9-like / B.1.1.33 + E484K”. In this sense, the detection of N.9 and “N.9-like / B.1.1.33 + E484K” reinforces the importance of continuous real-time genomic surveillance to monitor the emergence and behavior of the SARS-CoV-2 variants in the population.

Data availability

The SARS-CoV-2 genomes generated and analysed in this study are available at GISAID (https://www.gisaid.org/), under the ID: EPI_ISL_2086268 and EPI_ISL_2557401.
