# Supplementary material for: SARS-CoV-2 variant N.9 identified in Rio de Janeiro, Brazil
Source: Mem Inst Oswaldo Cruz. 2021 Nov 8;116:e210166. doi: 10.1590/0074-02760210166 (PMC8577066; doi:10.1590/0074-02760210166)
Supplement: Supplementary file 1 [file 1678-8060-mioc-116-e210166-s1.pdf]

Mem Inst Oswaldo Cruz, Rio de Janeiro, Vol. *116*: e210166, 2021

TABLE I

Polymerase chain reaction primers

Primers	Fragment length (bp)	Genome position*
CoV1F-CoV1R	1416	1-1416
CoV2F-CoV2R	1449	1204-2652
CoV3F-CoV3R	1408	2381-3788
CoV4F-CoV4R	1419	3505-4923
CoV5F-CoV5R	1449	4680-6128
CoV6F-CoV6R	1383	5924-7306
CoV7F-CoV7R	1418	7100-8517
CoV8F-CoV8R	1413	8321-9733
CoV9F-CoV9R	1383	9520-10902
CoV10F-CoV10R	1407	10697-12103
CoV11F-CoV11R	1395	11893-13287
CoV12F-CoV12R	1407	13085-14491
CoV13F-CoV13R	1412	14310-15721
CoV14F-CoV14R	1414	15523-16936
CoV15F-CoV15R	1405	16725-18129
CoV16F-CoV16R	1413	17913-19325
CoV17F-CoV17R	1431	19139-20569
CoV18F-CoV18R	1416	20370-21785
CoV19F-CoV19R	1406	21588-22993
CoV20F-CoV20R	1405	22796-24200
CoV21F-CoV21R	1372	23997-25368
CoV22F-CoV22R	1421	25165-26585
CoV23F-CoV23R	1423	26437-27859
CoV24F-CoV24R	1422	27653-29074
CoV25F-CoV25R	1011	28862-29872

*: genome position regarding reference genome sequencing (MT350282.1). F: forward primer; R: reverse primer.

CC BY

TABLE II

DNA sequencing primers

Primer name	Sequence	Genome position*
CoV 1F	5’ ATTAAAGGTTTATACCTTCCCAGGTAAC 3’	1-28
CoV 5′AR	5’ GTGTCCTGTCAACGACAGTAAT 3’	143-164
CoV 1AF	5’ CTGCACCTCATGGTCATGTTA 3’	498-518
CoV 5′BR	5’ CACCAAGTGTCTCACCACTAC 3’	561-581
CoV 1AR	5’GTTCCGTGTACCAAGCAATTT 3’	975-995
CoV 2F	5’ CCAAATGTGCCTTTCAACTCTC 3’	1204-1225
CoV 1R[c]	5’ AGACTATGCTCAGGTCCTACTT 3’	1395-1416
CoV 2AF	5’ GTGGAAACTGTGAAAGGTTTGG 3’	1727-1748
CoV 2AR	5’ GTCCACCGACAATTTCACAAG 3’	2232-2252
CoV 3F	5’ GGTGAAATATTTGTCACGCACTC 3’	2381-2403
CoV 2R	5’ GTGTCTTTGATTTCGAGCAACAT 3’	2630-2652
CoV 3AF	5’ TGTGGCAGATGCTGTCATAAA 3’	2887-2907
CoV 3AR	5’ ACAACTGGTGTAAGTTCCATCTC 3’	3302-3324
CoV 4F	5’ CAATGCCATGCAAGTTGAATCT 3’	3505-3526
CoV 3R	5’ AGTAGACATTTGTGCGAACAGTA 3’	3766-3788
CoV 4AF	5’ GAAGTTACAACAACTCTGGAAGAAA 3’	3977-4001
CoV 4AR	5’ CAAGTGGCATTGTAACAAGAGTTT 3’	4593-4616
CoV 5F	5’ CAGCTACAGTTTCTGTTTCTTCAC 3’	4680-4703
CoV 4R	5’ AAGGTGATAACTTCACCATCTAGG 3’	4900-4923
CoV 5AF	5’ GAGTACTACCACACAACTGATCC 3’	5162-5184
CoV 5AR	5’ AGGTGACTCCTGTTGTACTAGAT 3’	5652-5674
CoV 6F	5’ GTTGTTTGTACAGAAATTGACCCT 3’	5924-5947
CoV 5R	5’ GCTCTCTTGAAGCAGGTTTCT 3’	6108-6128
CoV 6AF	5’ AATCCTACCATACAGAAAGACGTT 3’	6425-6448
CoV 6AR	5’ CCGACACTCTTAACAGTATTCTTTG 3’	6861-6885
CoV 7F	5’ GCAACCTACTGTACTGGTTCTATAC 3’	7100-7124
CoV 6R	5’ CATGATTGCAGCCAATCCAA 3’	7287-7306
CoV 7AF	5’ GTGTTAATTGTGATACATTCTGTGC 3’	7620-7644
CoV 7AR	5’ ACATTGTCTAAGGACACATTCTTTG 3’	8127-8151
CoV 8F	5’ CGTGACCTTGGTGCTTGTAT 3’	8321-8340
CoV 7R	5’ ACAACTTGTCTAGTAGTTGCACAT 3’	8494-8517
CoV 8AF	5’ AGTCATAACAAGAGAAGTGGGTTT 3’	8833-8856
CoV 8AR	5’ ACAGCATCTACACCACAGAAA 3’	9316-9336
CoV 9F	5’ CCTTATGTCATTCACTGTACTCTGT 3’	9520-9544
CoV 8R	5’ GAACCAATAGAAATGCTTTGTGGA 3’	9710-9733
CoV 9AF	5’ CCAACCACCACAAACCTCTAT 3’	10015-10035
CoV 9AR	5’ CAACACTACCACATGAACCATTAAG 3’	10475-10449
CoV 10F	5’ GGAGACAGGTGGTTTCTCAATC 3’	10697-10710
CoV 9R	5’ CCCAATATGGTACGTCCATTCA 3’	10881-10902
CoV 10AF	5’ TCTCTTGCCACTGTAGCTTATT 3’	11192-11213
CoV 10AR	5’ GTCAGTCTAAAGTAGCGGTTGAG 3’	11663-11685
CoV 11F	5’ GCAACAACTCAGAGTAGAATCATC 3’	11893-11916
CoV 10R	5’ TGAGGCTATAGCTTGTAAGGTTG 3’	12081-12103

Primer name	Sequence	Genome position*
CoV 11AF	5’ CAATGCAAGAGATGGTTGTGTT 3’	12415-12436
CoV 11AR	5’ AAACCTACAAGGTGGTTCCAG 3’	12890-12910
CoV 12F	5’ GTAGATGCTGCTAAAGCTTACAAAG 3’	13085-13109
CoV 11R	5’ CCTTTAGGATTTGGATGATCTATGTG 3’	13262-13287
CoV 12AF	5’ AATTGTTGTCGCTTCCAAGAAA 3’	13594-13615
CoV 12AR	5’ TCACCGAAATCATACCAGTTACC 3’	14080-14102
CoV 13F	5’ GGATCAGACATACCACCCAAAT 3’	14310-14331
CoV 12R	5’ GCTCTCTGAAGTGGTATCCAG 3’	14471-14491
CoV 13AF	5’ ATGCTGCTATCAGCGATTATGA 3’	14780-14801
CoV 13AR	5’ AGGCATGGCTCTATCACATTT 3’	15301-15321
CoV 14F	5’ AACATTTGTCAAGCTGTCACG 3’	15523-15543
CoV 13R	5’ CGTCAGAGAGTATCATCATTGAGAAAT 3’	15695-15721
CoV 14AF	5’ TTAGCTATAGATGCTTACCCACTTAC 3’	16024-16049
CoV 14AR	5’ AAGTCAGTAACATTATCGCTACCA 3’	16530-16553
CoV 15F	5’ ACATCTTTCATGGGAAGTTGGTA 3’	16725-16747
CoV 14R	5’ GCATTACTGTATGTGATGTCAGC 3’	16914-16939
CoV 15AF	5’ GCGCTCGTGTAGAGTGTTT 3’	17246-17264
CoV 15AR	5’ GGAATTCTCTTACCACGCCTATT 3’	17712-17734
CoV 16F	5’ CAGATTTAATGTTGCTATTACCAGAGC 3’	17913-17939
CoV 15R	5’ GTCAACACTGAGGTGTGTAGG 3’	18109-18129
CoV 16AF	5’ TTTCCAGAGTTAGTGCTAAACCA 3’	18437-18459
CoV 16AR	5’ CAAGCCGCATTAATCTTCAGTTC 3’	18943-18965
CoV 17F	5’ TCTATTCTTATGCCACACATTCTGA 3’	19139-19163
CoV 16R	5’ GGTGTGTGGAATGCATGTTTAT 3’	19304-19325
CoV 17AF	5’ AAGGGACACTTTGATGGACAA 3’	19654-19674
CoV 17AR	5’ GGCTTCTCCAATTAATGTGACTC 3’	20111-20133
CoV 18F	5’ ACTGATTGGACTAGCTAAACG 3’	20370-20390
CoV 17R	5’ CCTTAGAAACTACAGATAAATCTTGGG 3’	20543-20569
CoV 18AF	5’ TGCTGGTTCTGATAAAGGAGTT 3’	20871-20892
CoV 18AR	5’ AGAATAGGAAGACAACTGAATTGGA 3’	21363-21387
CoV 19F	5’ CACTAGTCTCTAGTCAGTGTGTTAAT 3’	21588-21613
CoV 18R	5’ CTTTGGTCCCAGAGACATGTATAG 3’	21762-21785
CoV 19AF	5’ GAAAGTGAGTTCAGAGTTTATTCTAGTG 3’	22022-22049
CoV 19AR	5’ GCAAATCTGGTGGCGTTAAA 3’	22586-22605
CoV 20F	5’ CCAGGGCAAACTGGAAAGA 3’	22796-22814
CoV 19R	5’ GCTACCGGCCTGATAGATTTC 3’	22973-22993
CoV 20AF	5’ GTGATCCACAGACACTTGAGA 3’	23292-23312
CoV 20AR	5’ CTGCATTCAGTTGAATCACCAC 3’	23790-23811
CoV 21F	5’CAAGCAAGAGGTCATTTATTGAAGA 3’	23997-24021
CoV 20R	5’ CCGCTAACAGTGCAGAAGT 3’	24182-24200
CoV 21AF	5’ CCTTTCACGTCTTGACAAAGTT 3’	24502-24523
CoV 21AR	5’ GAGTCTAATTCAGGTTGCAAAGG 3’	24980-25002
CoV 22F	5’ AGAACTTGGAAAGTATGAGCAGTA 3’	25165-25188
CoV 21R	5’ TTGACTCCTTTGAGCACTGG 3’	25349-25368
CoV 22AF	5’ GTTTGTAACAGTTTACTCACACCTT 3’	25650-25674

Primer name	Sequence	Genome position*
CoV 22AR	5’ GTCGTCGTCGGTTCATCATAA 3’	26181-26201
CoV 23F	5’ CTGAATTCTTCTAGAGTTCCTGATCTT 3’	26437-26463
CoV 22R	5’ GTTCCATTGTTCAAGGAGCTTT 3’	26564-26585
CoV 23AF	5’ GTAATCGGAGCTGTGATCCTTC 3’	26937-26958
CoV 23AR	5’ CTCTTGGTAGTGATAAAGCTCACA 3’	27436-27459
CoV 24F	5’ TCATCAGACAAGAGGAAGTTCAAG 3’	27653-27676
CoV 23R	5’ TGCAGTTCAAGTGAGAACCA 3’	27840-27859
CoV 24AF	5’ CCAGGAACCTAAATTGGGTAGTC 3’	28163-28185
CoV 24AR	5’ CCGTCTTTGTTAGCACCATAGG 3’	28638-28659
CoV 25F	5’ TCAACTCCAGGCAGCAGTA 3’	28862-28880
CoV 24R	5’ TGCTTTAGTGGCAGTACGTTT 3’	29054-29074
CoV 25AF	5’ CCCACCAACAGAGCCTAAA 3’	29362-29380
CoV 25AR	5’ GAGTTTAGGCCTGAGTTGAG 3’	29518-29537
CoV 3’F	5’ CTTGAAAGAGCCACCACATT 3’	29708-29727
CoV 25BR	5’ CTCTTCCATATAGGCAGCTCTC 3’	29779-29800
CoV 25R	5’ TTGTCATTCTCCTAAGAAGCTA 3’	29851-29872

*: genome position regarding reference genome sequencing (MT350282.1). F and AF: forward primers R; AR and BR: reverse primers.
